# PMUT-Based System for Continuous Monitoring of Bolted Joints Preload

**DOI:** 10.3390/s24134150

**Published:** 2024-06-26

**Authors:** Stefano Sanvito, Marco Passoni, Domenico Giusti, Marco Terenzi, Carlo Prelini, Monica La Mura, Alessandro Stuart Savoia

**Affiliations:** 1 Department of Engineering and Applied Science, University of Bergamo, Via Galvani, 2, 24044 Dalmine, Italy; 2 STMicroelectronics, Via C. Olivetti 2, 20864 Agrate Brianza, Italy; 3 STMicroelectronics, Via Tolomeo 1, 20007 Cornaredo, Italy; 4 Department of Industrial, Electronic and Mechanical Engineering, Roma Tre University, Via della Vasca Navale 84, 00146 Roma, Italy

**Keywords:** piezoelectric micromachined ultrasonic transducer, PMUT, MEMS, ultrasound, non-destructive testing, structural health monitoring, bolted joint, bolt preload, finite elements method

## Abstract

In this paper, we present a bolt preload monitoring system, including the system architecture and algorithms. We show how Finite Element Method (FEM) simulations aided the design and how we processed signals to achieve experimental validation. The preload is measured using a Piezoelectric Micromachined Ultrasonic Transducer (PMUT) in pulse-echo mode, by detecting the Change in Time-of-Flight (CTOF) of the acoustic wave generated by the PMUT, between no-load and load conditions. We performed FEM simulations to analyze the wave propagation inside the bolt and understand the effect of different configurations and parameters, such as transducer bandwidth, transducer position (head/tip), presence or absence of threads, as well as the frequency of the acoustic waves. In order to couple the PMUT to the bolt, a novel assembly process involving the deposition of an elastomeric acoustic impedance matching layer was developed. We achieved, for the first time with PMUTs, an experimental measure of bolt preload from the CTOF, with a good signal-to-noise ratio. Due to its low cost and small size, this system has great potential for use in the field for continuous monitoring throughout the operative life of the bolt.

## 1. Introduction

Bolted joints are a fundamental element in engineering applications, providing a secure yet detachable means for assembling components [1]. The performance of these joints critically depends on the clamping force exerted by the bolt, which, if improperly applied or maintained, can lead to mechanical failure through loosening or fatigue of the joint components [2]. Therefore, ensuring the proper clamping force throughout the joint’s operative life is essential for structural integrity in various mechanical systems.

Several methods have been proposed and used to apply and measure the preload of bolts. The most common methods include torque control, angle control, bolt elongation measurement, direct tension indicators, strain gauges, and Ultrasonic Testing (UT). Torque control involves measuring the torque during the application of tension and stopping when a certain value is reached. Although torque can be easily measured with a torque wrench, preload accuracy may be low due to difficulties in accurately determining friction coefficients [3,4,5]. Moreover, torque control is unsuitable for preload monitoring. Angle control consists in turning the nut to a specific angle that corresponds to a known elongation and, therefore, to a certain preload. However, this method also has low accuracy, as the turn angle depends on many parameters and may need to be determined empirically [3]. Bolt elongation measurement requires access to both ends of the bolt with a micrometer before and after tension is applied [3]. This requirement makes the method impractical in real-world scenarios where such access is often restricted or entirely unavailable. Direct tension indicators, typically washers with bumps designed to flatten at a certain load, have the disadvantage of being specific to a particular joint and are unsuitable for monitoring the bolt during its operative life [6]. The strain gauge method requires an electronic or piezoelectric component specifically placed to change electrical impedance proportionally to the applied preload. This can occur through compression (washer-like) or attachment parallel to the bolt axis [6]. Ultrasonic Testing, particularly the method based on the acousto-elastic effect, leverages the fact that internal stress changes both the sound propagation speed and the bolt length. Both these phenomena lead to an increase in sound propagation time along the axis, with respect to the no-load condition, thereby causing a change in the time-of-flight (CTOF) proportional to the internal stress [7]. The CTOF can be measured by an ultrasonic transducer attached to one end of the bolt, thus operating in pulse-echo mode. Ultrasonic Testing is recognized as a high-accuracy mean for monitoring the clamping force in bolted joints [3,8,9]. UT is typically performed temporarily during the joint inspection, even if the transducer itself may be a very low-cost disposable component. That is because the equipment needed to operate the transducers is expensive and requires a skilled operator. Recently, inspired by the Internet of Things (IoT) paradigm, researchers have explored the possibility of equipping bolts with an embedded system for continuous preload monitoring. This advancement has been facilitated by adopting technological solutions that ensure a high degree of integration, very low power consumption, and significantly reduced costs. One such system, described in [10,11], employs traditional ultrasonic transducers—namely, piezoelectric ceramics; however, these suffer from limited integration potential.

In recent decades, Piezoelectric Micromachined Ultrasonic Transducers (PMUTs) have gained attention because of their potential for low cost and miniaturization due to their compact dimensions and possibility of integration with CMOS electronics [12]. Despite these advantages, challenges arise in effectively coupling PMUTs to solid materials. While effective operation through air-coupling [13,14] and liquid-coupling [15] has been demonstrated, solid coupling presents difficulties due to the acoustic impedance mismatch between the transducer and medium. PMUT coupling to solid material has been experimentally demonstrated using an interposed liquid medium [16], but a fully solid (“dry”) coupling would be preferable for industrial applications.

In this paper, we introduce a PMUT-based bolt preload measuring system designed for low-cost, low-power operation and intended for continuous monitoring during the entire operational lifespan of the bolt. This system features an array of PMUTs that are dry-coupled to the bolt surface using a novel assembly process. Previous studies [17,18] have demonstrated the feasibility of this approach through simulation. In this work, we propose an improved design of the PMUT array that operates at a higher frequency. We experimentally implemented the entire system using a dedicated analog front-end (AFE) transceiver application-specific integrated circuit (ASIC) for the PMUT transmit (TX) and receive (RX) functions, paired with an analog-to-digital converter (ADC) and a microcontroller unit (MCU) that manages the signal processing in a low-cost and low-power configuration. Through experimental characterization, we show that custom-designed signal processing algorithms can address some of the limitations associated with the use of low-power electronics while requiring only minimal computational resources. As a result, the proposed system offers high accuracy, minimal impact on the joint design, low cost, and compact size. The design process of the measurement setup and operating conditions was aided by Finite Element Method (FEM) simulations of wave propagation within the bolt, which provided additional insights for future improvements.

The paper is organized as follows: Section 2 details the specific features of the PMUT used in this work, including its assembly and coupling to the bolt, the architecture of the measuring system, the experimental setup, and the simulation environment. Section 3 presents the simulation results and their role in the system design, along with the experimental results. Section 4 discusses the results and offers final remarks.

## 2. Materials and Methods

### 2.1. PMUT Design and Microfabrication

We designed a 12-element, 2D PMUT array to operate in immersion around 2 MHz with broadband response [19]. The 12 array elements, each hexagonal in shape, are arranged on a regular hexagonal grid with a pitch, $p_{e}$, of 862 µm, which is the center-to-center distance between two adjacent elements. Each array element is composed of 27 circular PMUT cells with a diameter of 145 µm, electrically connected in parallel, and arranged on a regular hexagonal grid with a pitch of 166 µm between the centers of two adjacent cells. The PMUT cell microstructure consists of a 4 µm thick silicon flexural plate, on which a 2 µm thick piezoelectric layer is deposited and patterned in a 108 µm diameter disk shape. The flexural plate is suspended over a cylindrical cavity created by back-etching the 400 µm thick silicon microfabrication substrate.

**Figure 1:**
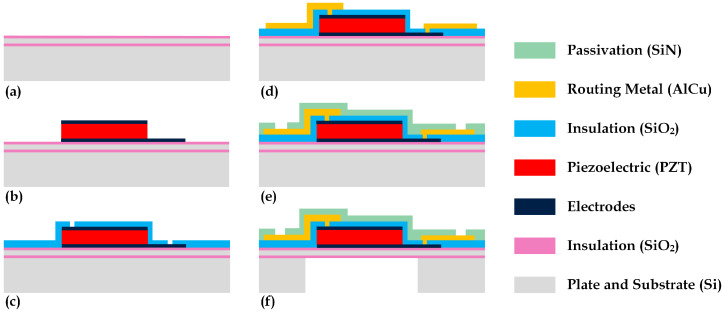
Description of the PMUT microfabrication process: creation of the silicon plate (**a**); deposition and patterning of the piezoelectric stack (**b**), isolation layer (**c**), and routing metal (**d**); device passivation and interconnection pads opening (**e**); substrate thinning and etching of the cavities (**f**).

The fabrication of the 2D PMUT array employed a sol–gel lead zirconate titanate (PZT) thin-film-based MEMS technology developed by STMicroelectronics [20], with the main process steps outlined in Figure 1. Initially, a 4 µm thick elastic plate layer comprising an oxide–silicon–oxide stack was created on a silicon wafer, as shown in Figure 1a. This was followed by the deposition and patterning of the bottom electrode, a 2 µm thick sol–gel PZT layer, and the top electrode, depicted in Figure 1b. Subsequently, the structure was insulated using SiO_2_ and an AlCu routing metal was applied and patterned, as detailed in Figure 1c and Figure 1d, respectively. After having deposited a final SiN passivation layer partially etched to open the interconnection pads, shown in Figure 1e, the thickness of the wafer was reduced to 400 µm and cavities were formed by backside etching of the silicon, as illustrated in Figure 1f.

**Figure 2:**
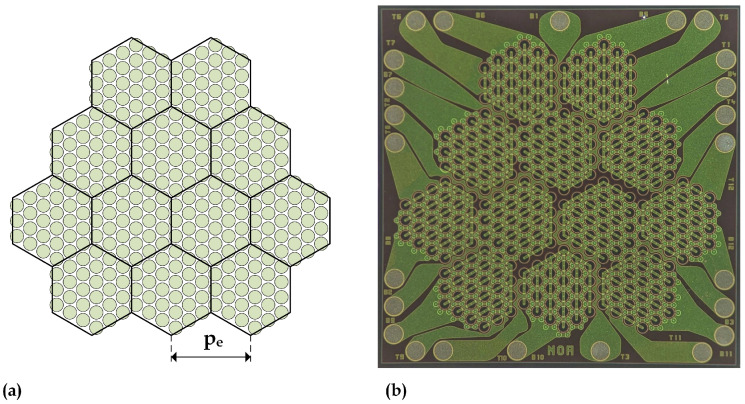
Layout of the 12-element 2D PMUT array (**a**) and fabricated die (**b**).

Figure 2a shows the layout of the 2D PMUT array, where the 12 hexagonal elements and the arrangement of the cells within each element are visible. For each element, we routed the top and bottom electrode contacts to the area surrounding the device using routing metal, enabling individual electrical accessibility for each array element. This organization allows the use of the entire array or only a subset of the array, accessing any number of elements in either TX or receive RX, even independently. This flexibility allows us to adjust the relative aperture sizes and, by changing the type of electrical connection, i.e., parallel or series, to change the electrical impedance and sensitivity of the transducer to better match the electrical characteristics of the analog front-end circuits. The result is optimized performance in terms of TX efficiency and RX signal-to-noise ratio (SNR).

Figure 2b shows a photograph of the fabricated die, which is square with a side length of 4.5 mm. Aluminum–copper (AlCu) metallization was used to connect the top and bottom electrodes to the circular pads along the edges of the die.

### 2.2. PMUT Assembly Process

**Figure 3:**
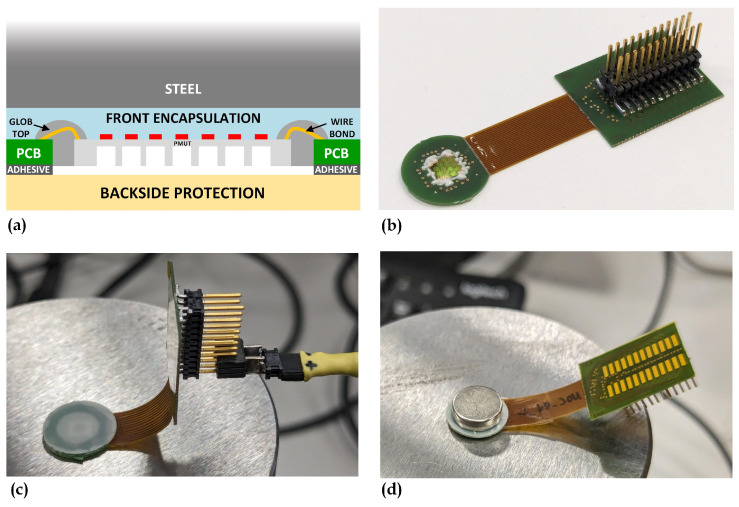
Schematic diagram of the PMUT packaging approach (**a**). PMUT array bonded to the rigid flex PCB (**b**). PMUT assembly permanently attached (**c**) and magnetically pressed (**d**) onto a steel block.

The device assembly approach is schematically illustrated in Figure 3a. The PMUT array was electrically and mechanically connected to a rigid-flex printed circuit board (PCB), consisting of two 0.4 mm thick rigid FR4 sections, through a 0.1 mm thick flexible polyimide section. One rigid section of circular shape houses the PMUT array die in a square opening located at the center. The other rigid section accommodates a 24-pin connector enabling electrical access to the top and bottom electrodes of the array elements. Electrical interconnection between the die and the PCB was made by Au wire wedge bonding, achieving wire bond heights below 100 µm thanks to the coplanar alignment of die and PCB surfaces. Following wire bonding, a glob-top epoxy resin, Epotek 301 (Epoxy Technology Inc., Billerica, MA, USA) filled with Al_2_O_3_ powder was applied to encapsulate and protect the bonds while also filling the gap between the die and the PCB, thereby providing mechanical continuity. Figure 3b shows a photograph of the PMUT die assembled on the PCB through wire bonding and glob-top encapsulation.

An acrylic adhesive layer was then applied to the back of the PCB to which a 200 µm thick Mylar plastic sheet was attached to protect the back of the die and provide mechanical support for the entire assembly.

It is important to note that the glob-top applied around the die provides mechanical support but decouples it from the backside protection layer, effectively reducing the mass of the PMUT mechanical support. This results in a higher Q response, which provides better electro-acoustic matching [21]. Moreover, decoupling the die from the package makes it less susceptible to potential mechanical instabilities of the package itself, such as those caused by temperature variations.

A front encapsulation layer was then applied to the surface of the assembly for mechanical and acoustic coupling of the PMUT to the propagation medium (steel in this case). This was achieved by a thin layer of a soft solid material consisting of a polyurethane elastomer with Shore A hardness of 20, a specific acoustic impedance of 1.44 MRayl, and a bulk speed of sound of 1386 m/s. The thickness of this layer was conveniently selected to control its acoustic resonant effect caused by multiple reflections at the PMUT surface and at the interface between the front encapsulation and steel interface. Compatibly with the minimum thickness of 300 µm allowed by the proposed assembly technique, the front encapsulation layer thickness was set to 650 µm to achieve the first 3 thickness modes in the PMUT frequency band.

The mechanical and acoustic coupling between the front encapsulation layer and steel was achieved using the polyurethane elastomer itself as an adhesive through direct application and, alternatively, via pressure adhesion by placing a magnet on the back of the packaged device. In this work, we successfully tested both approaches, by permanently attaching (Figure 3c) and magnetically pressing (Figure 3d) the PMUT to a polished AISI 4340 steel block. Although the permanent attachment represents a more attractive solution for industrialization, for practicality, we used magnetic pressing to couple the PMUT with the bolt in the implementation of the measurement system setup.

**Figure 4:**
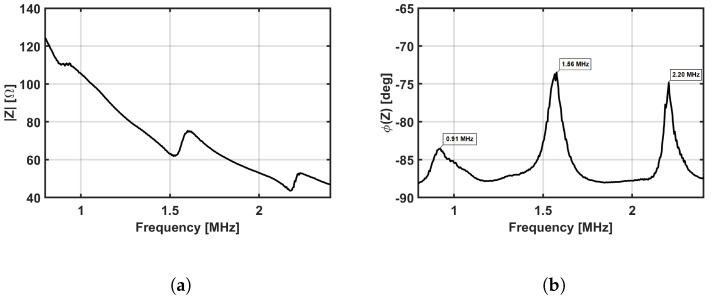
(**a**,**b**) Electrical impedance measurements of the 3 central array elements connected in parallel. The first 3 thickness modes resonance frequencies of the front encapsulation layer are 0.91 MHz, 1.56 MHz, and 2.20 MHz.

Electrical impedance measurements of the PMUT were carried out to accurately assess the resonance frequencies of the PMUT coupled to the front encapsulation layer, which were employed to determine the optimal frequency of the TX driving signals used for the characterization of the measurement system described later. Figure 4a,b show the electrical impedance of the three central PMUT array elements connected in parallel measured in the range 0.8–2.4 MHz. The impedance phase curves (Figure 4b) revealed three peaks at 0.91 MHz, 1.56 MHz, and 2.20 MHz, corresponding to the first three resonant coupled modes of the PMUT, vibrating in flexural mode, and the front encapsulation layer, vibrating in thickness mode. These three coupled modes are identified by the three phase resonant peaks. The second peak, in particular, had the smallest phase angle because it was the closest to the fundamental PMUT resonant mode and, therefore, it was expected to provide the highest coupling efficiency. Thus, the second mode was selected as the optimal frequency for operation.

### 2.3. Simulation of the Propagation of Acoustic Waves in the Bolt

Understanding how acoustic wave propagates inside the bolt is key to design a sound CTOF measuring system. Performing experiments about this phenomena is hardly feasible because of the loading effect. Therefore, Finite Element Method (FEM) simulations are usually preferred. We have conducted FEM simulations using COMSOL Multiphysics^®^. These simulations provide a visual representation of wave behavior under different operating conditions and geometries of the bolt, as well as by varying characteristic parameters of the transducers. The performed finite element analyses are aimed at informing the optimization of transducer placement and understanding which parameters have greater influence on the quality of the output signal.

Considering the whole measuring system, we are interested in the behavior of the transducers at the electrical port, i.e., to use the voltage signal generated by the AFE as input for the simulation model, and the voltage signal at RX transducers terminals as output, connected to RX AFE. To do so, the most accurate approach would be to model both the transducers and the bolt with finite elements, as performed in [18]. This approach leads to high-accuracy results but is time-consuming, both for modeling and for simulation running time. Instead, we choose a simplified approach, with less accuracy but much faster simulation time. The simulation procedure is the following. Firstly, a PMUT lumped elements model is used to compute the average velocity at the surface from the applied voltage; this is performed in Matlab and requires negligible computational resources. Then, the computed velocity is given as input to the actual finite element model and the velocity value at the RX transducers surface is taken as output. Finally, the same lumped elements model used before may be used to convert received velocity into voltage at PMUT terminals. This approach makes it possible to use single COMSOL physics, thereby requiring a short simulation time.

**Figure 5:**
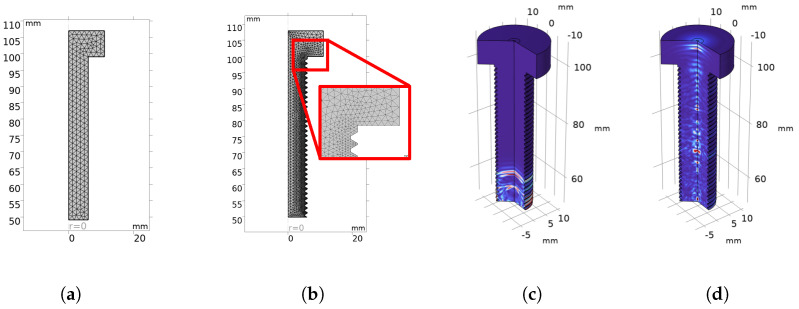
Mesh applied to the model and frames of the propagation of acoustic waves inside the bolt: (**a**) mesh of the unthreaded case, (**b**) mesh of the threaded case, (**c**) velocity map at t=3.20 µs, and (**d**) velocity map at t=11.0 µs.

The physics applied to the model is Acoustics → Ultrasound → Elastic Wave, Time Explicit. The simulation was carried out on a 2D axisymmetric representation of the bolt. Figure 5 shows the used geometry, representing an M12 bolt with a head diameter of 22 mm and a total length of L=58 mm, which is half the length of the one that was used for experimental tests, to reduce simulation time. The transducer is modeled as a segment placed on the head or the tip of the bolt.

Figure 5a,b show the mesh on a 2D axisymmetric representation, whose maximum element size is λ/2, while the element order is quartic [22].

A very popular model to represent a piezoelectric transducer with an electrical-equivalent lumped elements model is the Butterworth–Van-Dyke model, which can be conveniently adapted also to PMUTs [23]. By rearranging the electrical network equation and normalizing, the Transfer Function (TF) from applied voltage to the velocity at the surface of the transducer, which is proportional to the emitted pressure, can be boiled down to a damped harmonic oscillator, which can be written in the Laplace domain as Equation (1)

(1) $$U\left(s\right)=\frac{1}{s^{2}+2\zeta \omega_{0}s+\omega_{0}^{2}}V\left(s\right)$$

where U(s) and V(s) are the velocity and voltage signals, respectively; ζ is the transducer damping ratio; and $\omega_{0}$ is the transducer natural frequency, which is related to the undamped resonance frequency $f_{0}$ by $\omega_{0}=2\pi f_{0}$. The values of damping and resonance parameters of $f_{0}=1.6$ MHz and ζ=0.03 were estimated by fitting the electrical impedance measurement of the PMUT attached to a stainless steel block. We use a normalized version of the harmonic oscillator equation because our purpose is to show qualitative behavior of wave propagation; therefore, the absolute gain of the TF is not relevant. For this reason, the simulation outputs reported in the following sections of the article will have the velocity in Arbitrary Units (A.U.).

The voltage input signal is processed through the sensor’s TF (Equation (1)) to produce the time-domain velocity signal expected on the emitter’s surface. This velocity signal is then imposed as a boundary condition on the emitters within the finite element model, allowing us to execute a transient analysis. Therefore, the emitter is modeled as a baffled piston. The same TF can be used dually for RX section, with incident velocity as the input and voltage at electrodes as output.

The bolt material is Steel AISI 4340, with density ρ=7850 kg/m^3^, sound propagation speed *c* = 5856 m/s, Young’s modulus $E=205\times 10^{9}$ [Pa], and a Poisson’s ratio ν=0.28. In a pulse-echo scenario, with no clamping force applied, the first echo (i.e., the first one after the reflection from the opposite side) is expected after t=2L/c = 19.8 µs.

An example of the resulting propagation of acoustic waves inside the bolt is shown in Figure 5. In particular, Figure 5c shows the emitted acoustic wave just after emission, propagating upwards, and Figure 5d shows the reflection at the bolt head.

Several simulations were carried out in order to assess the impact of the transducer’s position and bandwidth, the relevance of the presence of threads, and the effect of operating frequency. In some of the simulations, we introduced simplified conditions, for example, an unthreaded shank, a low reflecting boundary around the shank, and a wide bandwidth transducer, which are instrumental for a better understanding of the complex wave propagation phenomena that take place inside the bolt. Specifically, the simulations were performed as follows:

- The transducer was moved from head to tip of the bolt to support the design of the measurement setup by choosing the best transducer’s position in terms of output signal quality;
- The shank boundary was then set to reflective to investigate the different types of waves propagating inside the bolt;
- The transducer bandwidth was narrowed to better represent the physical device and evaluate the effect on the propagating waves;
- The threads were introduced in the bolt model to evaluate to what extent their presence affects the output signal;
- The operating frequency was increased from 1.6 MHz to 7 MHz to investigate whether increasing the frequency could yield appreciable improvements in signal reading and processing.

### 2.4. System Architecture

The system is composed of two boards: the PMUT Board and Control Board. The Control board is a custom board designed by us and has the following parts: a monolithic AFE, an ADC, an MCU, and a power management subsystem. The Control Board dimensions are 60×60 mm.

The AFE is a monolithic ASIC [24,25], comprising a transmitter (TX) section with a high-voltage pulser, a receiver (RX) section with a low noise amplifier and a voltage buffer, and a T/R switch to switch the connected transducer between the transmitter and the receiver. This is the typical AFE architecture used in high-end ultrasound applications, e.g., in medical ultrasound [26]. This AFE+ADC architecture allows maximum flexibility for signal processing, particularly the possibility to extract information from noisy signals. The AFE is designed for analog signals with frequency up to 10 MHz; its digital lines to control the TX transistors have a maximum refresh rate of 20 MSa/s.

The ADC is single-channel, operated at 20 MSa/s acquisition frequency.

The MCU belongs to the STM32U5 family, which allows both low-power operation and capability to simultaneously drive the Transmitter digital input lines at 20 MHz and read the ADC digital output at 20 MHz. We choose to use the 20 MHz time-base because it allows having low cost and complexity while still being able to measure CTOF with good accuracy. In fact, despite a sampling time of 50 ns, thanks to proper signal processing, we can reach a CTOF resolution of a few nanoseconds (Section 3.2), which is compliant with the requirements for this application [18,27].

### 2.5. Experimental Setup

**Figure 6:**
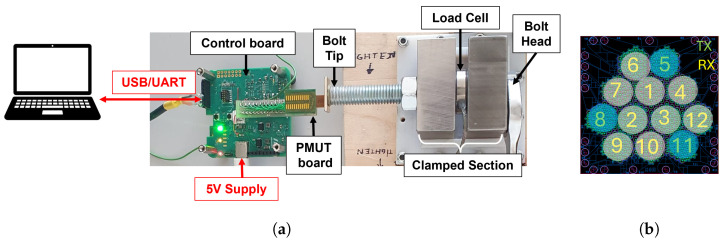
(**a**) Experimental setup comprising Control board, PMUT board, reference load cell, and bolted joint; (**b**) TX-RX connection of PMUT elements.

Figure 6 shows the experimental setup. The PMUT board is kept in contact with the bolt tip with a magnet. The measurement is performed in pulse-echo mode, i.e., the transmitter and receiver transducers are on the same side of the bolt, and acoustic wave is picked up by the receiver after a reflection on the opposite side of the bolt. The joint is made with two steel blocks, each 20 mm thick. One of them is fixed to the base; the other is free to move along the direction of the bolt axis. Between the two joint parts there is a washer-shaped load cell, used as reference for the clamping force measure. The load cell model is LCM901-13-130KN, manufactured by Omega Engineering Inc., Norwalk, CT, USA. Its full scale is 130 kN. The bolt has hexagonal head and total length L=108 mm, accounting for the shank, the thread, and the head. The thread size is M12. A single nut is tightened to create the preload. The clamped section length is Lc=47 mm. The head and the tip of the bolt were reworked with Electrical Discharge Machining to decrease the roughness of the surface and improve acoustic signal reflection.

The typical connection of transducer elements to the AFE is with all elements in parallel, used for both TX and RX [28]. We choose instead to connect the twelve PMUT elements, as shown in Figure 6b, to minimize cross-talk effects: three external elements are used as transmitters, to narrow the emitted beam as much as possible, and nine elements are used as receivers.

### 2.6. Measurement Process

Each CTOF measure consists of the following sequence of operations. Firstly, the excitation signal is applied to the transmitter elements and, simultaneously, the voltage at the receiving elements is acquired from the ADC output. The excitation signal is a 32 V_pp_ 3-cycle square wave at 1.6 MHz, which was the closest frequency to the 1.56 MHz resonance of the PMUT that could be generated by the MCU with the current hardware and firmware configuration. The acquired data are then band-pass filtered to remove the out-of-band signals and noise. Then, the time delay between a pre-defined time-threshold and the nearest following zero-crossing is measured. Finally, the corresponding no-load delay is subtracted from the obtained delay—this is the CTOF measure, which is proportional to the preload. In order to have a useful measure, the CTOF, which is a time, needs to then be converted into preload (or stress) by multiplication with a factor, which is dependent on joint geometry, bolt features, and acoustic parameters, according to Equation (2)

(2) $$\sigma =\frac{cE}{2UL_{c}}CTOF$$

where σ is the axial stress, *c* is the propagation speed inside the bolt, *E* is the Young’s modulus of bolt material, $L_{c}$ is the clamped length, and *U* is the ultrasonic constant (i.e., the ratio between the ultrasonic elongation and the actual elongation). If $L_{c}\gg D$, where *D* is the bolt diameter, *U* does not depend on joint geometry, only on bolt material [27]. The use of Equation (2) requires that the joint parts, including the load cell, are much more rigid than the bolt, i.e., the whole deformation induced by the clamping force happens in the bolt, while the joint parts remain not deformed. Moreover, the condition $L_{c}\gg D$ allows to approximate the value of $L_{c}$ with the actual clamped length by assuming that the stress along the bolt axis is constant in the clamped length and zero everywhere else. Otherwise (for example, see results in [18,27]), a correction factor shall be applied to either *U* or $L_{c}$.

## 3. Results

### 3.1. Simulations

In this section, we show the results of the different transient analyses performed. In particular, for each case considered, we report the time-domain average axial velocity, filtered around the operating frequency, and the spatial distribution of the velocity magnitude at a fixed time. As can be noticed, the signal emitted by the transducer during transmission is also observed during reception, due to self-coupling of the transducer, in all the explored conditions. However, the signal rings down before reception of the first echo; therefore, no issue is expected from this cross-talk phenomenon. The transducer’s position, bandwidth, presence of threads, and operating frequency, set for each of the obtained images, are reported in Table 1.

**Table 1:** Summary of parameters of the conditions reported in Section 3.1.

	Figure 7	Figure 8	Figure 9	Figure 10	Figure 11	Figure 12
Positioning	HEAD	TIP	TIP	TIP	TIP	TIP
Shank boundary	Low-reflecting	Low-reflecting	Free	Free	Free	Free
Transducer bandwidth	Wide	Wide	Wide	Narrow	Wide	Wide
Threads	NO	NO	NO	NO	YES	NO
Frequency	1.6 MHz	1.6 MHz	1.6 MHz	1.6 MHz	1.6 MHz	7 MHz

**Figure 7:**
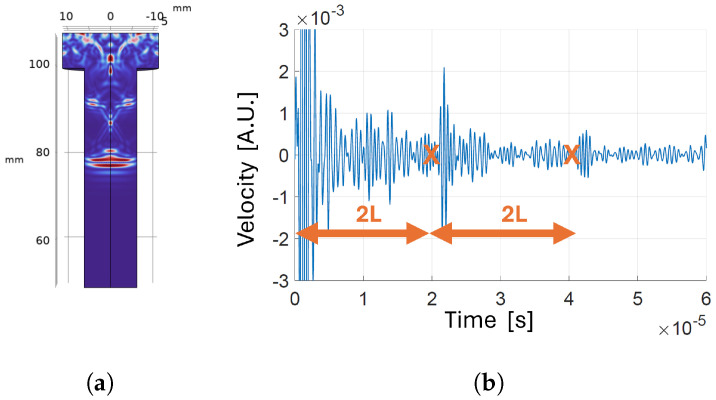
Transducer positioned on the head of the bolt with low-reflecting boundary around the shank: (**a**) velocity map a t=6.7 µs; (**b**) time-domain velocity signal at the receiver.

**Figure 8:**
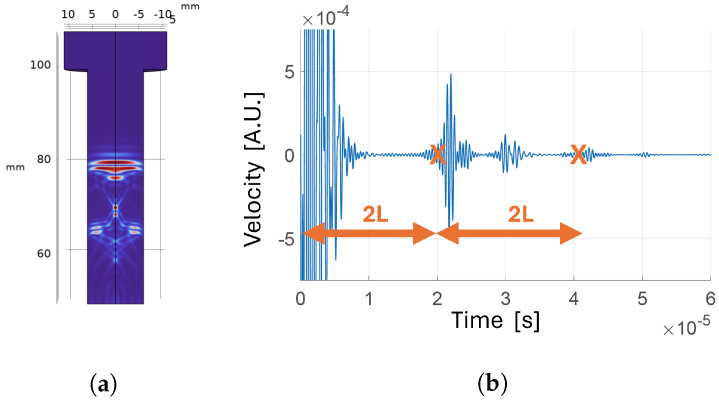
Transducer positioned on the tip of the bolt with low-reflecting boundary around the shank: (**a**) velocity map a t=6.7 µs; (**b**) time-domain velocity signal at the receiver.

**Figure 9:**
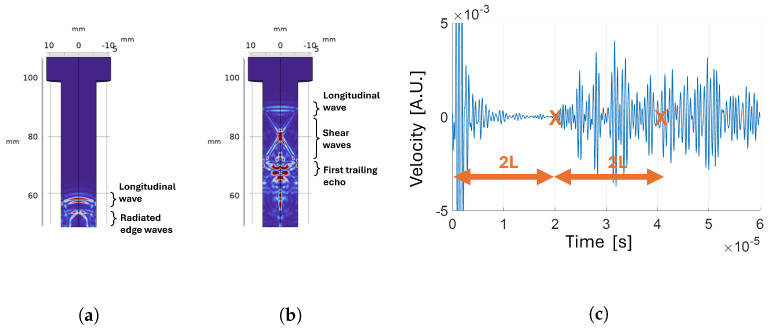
Transducer positioned on the bolt tip with reflecting boundary around the shank: (**a**) velocity map a t=3.1 µs; (**b**) velocity map a t=8.4 µs; (**c**) time-domain velocity signal at the receiver.

**Figure 10:**
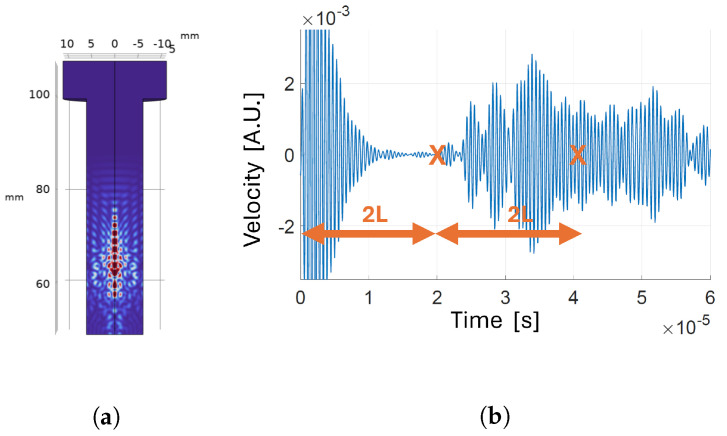
Transducer positioned on the tip and of the bolt, with reflecting boundary around the shank and narrow transducer bandwidth: (**a**) velocity map a t=6.7 µs; (**b**) time-domain velocity signal at the receiver.

**Figure 11:**
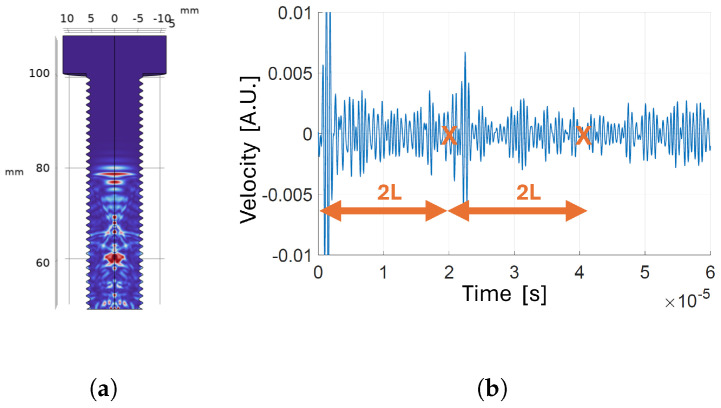
Transducer positioned on the tip and of the bolt, with threads: (**a**) velocity map a t=6.7 µs; (**b**) time-domain velocity signal at the receiver.

**Figure 12:**
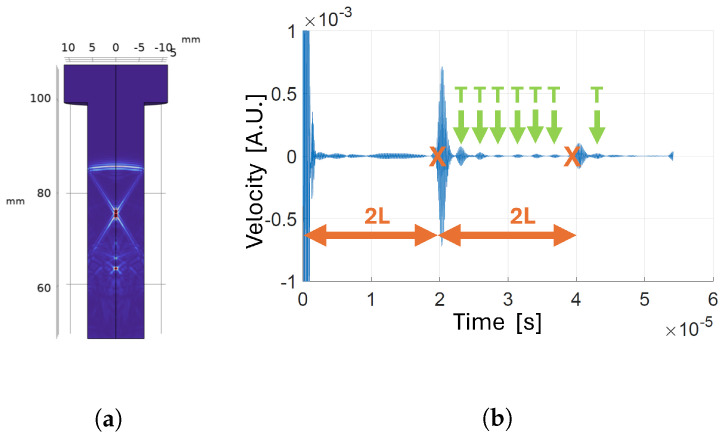
High -frequency transducer positioned on the tip of the bolt: (**a**) velocity map a t=6.7 µs; (**b**) time-domain velocity signal at the receiver.

#### 3.1.1. Position of the Transducer

To evaluate the impact of transducer placement on the effectiveness of first echo detection from the acquired signal, we used finite element analysis to determine the optimal position of the PMUT by relocating the transducer along the bolt’s axis from head to tip. The boundary type around the shank was set to “low-reflecting” in order to avoid masking of the reflections that take place inside the bolt head by contributions from other reflective boundaries. Figure 7b and Figure 8b show the time-varying velocity signal received by the transducer positioned on the bolt head and tip, respectively. The echo signal received after reflection from the opposite side is clearer when the transducer is positioned on the tip rather than on the head. In fact, as can be seen in Figure 7a, multiple reflections coming from the outer borders of the bolt head superimpose before and after arrival of the first echo, affecting its detectability. Therefore, it is preferable to locate the transducer on the bolt tip to effectively measure the CTOF.

#### 3.1.2. Analysis of Wave Propagation

Once the optimal position of the transducer was set, we set the boundary type around the shank as reflective to achieve a better understanding of waves propagation inside the bolt during experiments. Figure 9 shows the velocity magnitude maps in the bolt cross-section at different moments in time. When the pressure wave generated by the transmitter interacts with the boundary, part of the energy is converted in a shear wave, which travels towards the center of the bolt. When the shear wave reaches the opposite side, another mode conversion happens such that part of the energy is converted into a new pressure wave that travels in the same direction as the first one. This longitudinal wave is called a trailing echo. After the first trailing echo, the shear wave is reflected again and the process repeats, generating further trailing echoes, with each being less energetic than the previous. The delay between trailing echoes corresponds to the ratio between the bolt diameter and the shear wave velocity. This behavior is consistent with results reported in previous works, both in simulation [29,30] and experiments [31,32]. In Figure 9a, another type of wave is distinguishable: these are radiated shear waves generated by mode conversion on the bolt tip, at the boundaries of the transducer [30]. These additional waves originating from mode conversions at the reflective boundaries take away energy from the longitudinal wave, which generates the echo needed for the CTOF measurement. Nevertheless, experiments confirm that, in the developed system, spurious waves do not compromise the capability to distinguish the time of arrival of the signal of interest.

#### 3.1.3. Transducer Bandwidth

PMUTs working in solid-coupled conditions have a narrow bandwidth. To assess the effect of working with signals with limited bandwidth, we simulated the propagation of an acoustic wave generated by a more narrowband pressure signal. Figure 10 shows results of the simulation conducted by applying a narrowband signal. Comparing Figure 10a with Figure 9b, the different wave types are not clearly distinguishable anymore. While the wave types are not distinct, they are still present, while superimposed; therefore, the validity of measuring the CTOF at the time of the expected first echo still holds, as demonstrated with experiments in Section 3.2.

#### 3.1.4. Presence of Threads in the Bolt

The presence of threads in the bolt may, in principle, have an impact on wave propagation and on the correct detection of the TOF. The most relevant figure that impacts how the waves reflect is the ratio between the wavelength, λ=3.7 mm, and the dimension of the boundary surface roughness. In this case, we can take the bolt pitch p=1.75 mm as the representative figure of the surface roughness. When the wavelength is much higher than the roughness, the reflection is specular, and the behavior is the same as that of a smooth surface; on the other hand, when the wavelength is of the same order of magnitude of the roughness, phenomena of diffuse reflection arise [33]. As shown in Figure 11, the presence of threads generates additional noise in the received signal. However, with respect to the smooth shank case (Figure 9), the additional noise does not modify the fact that the first longitudinal echo reaches the receiver before the other wave types; therefore, the validity of the CTOF measure still holds.

#### 3.1.5. High Frequency Transducers

Conventional bulk-piezoelectric transducers for UT are typically operated in thickness mode in the 1–10 MHz frequency range [34,35]. The higher part of the frequency range is preferred when directivity and resolution are to be maximized, at the expense of a wide field of view [36], which is the case for bolt preload monitoring. Although manufacturing PMUTs that operate efficiently in such a high frequency range can be challenging and expensive, we performed a simulation in this frequency range ($f_{0}=7.0$ MHz) to evaluate the possible advantages of increasing the operating frequency and, consequently, directivity. The result of exciting the wide-aperture transducer placed at the bolt tip with a 7 MHz input signal is shown in Figure 12. As can be noticed, the stronger directivity results in clearer and more easily distinguishable echoes: the amplitude of the first and second echoes is much greater than the amplitude of trailing echoes. Therefore, increasing the frequency would enhance detection of the quantity of interest.

### 3.2. Experimental Measurements

**Figure 13:**
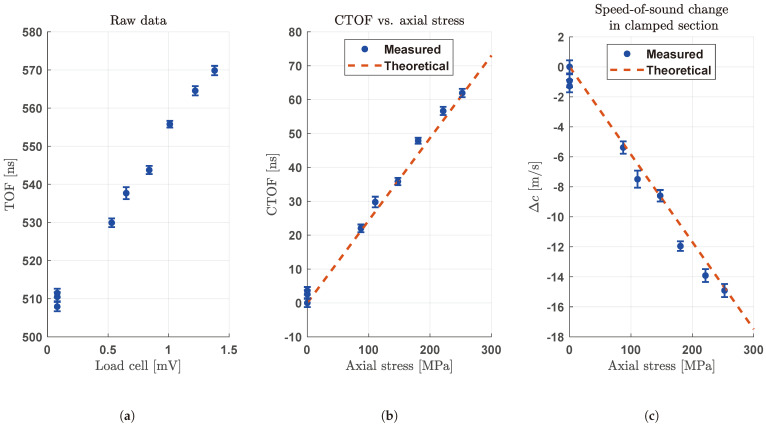
Experimental measurements; bars represent the standard deviation of repeated measurements. (**a**) Raw data: measured TOF vs. reference force measured with the load cell. (**b**) Conversion of raw data to CTOF vs. stress, and comparison with theoretical values. (**c**) Derived change in speed of sound due to acousto-elastic effect.

We performed experimental measurements with the setup described in Section 2.5 and according to the procedure described in Section 2.6. Based on the simulation results presented in Section 3.1, the transducer was positioned at the bolt tip, the selection of transmitting elements was conducted with the aim of increasing the aperture size, and the excitation was provided at low frequency (f=1.6 MHz). The output of this process is the CTOF. We changed the bolt preload manually, reaching a stress value of up to about σ=250 MPa, in order to test the whole elastic region before yield. Figure 13a shows the TOF, i.e., the time interval of the first zero crossing after a threshold set to t=36.9 µs, corresponding to the nominal time when the first echo is expected. To evaluate the repeatability, we performed repeated measurements at constant preload—in particular, for each point in Figure 13a, we performed 20 measures on the same bolt and without repositioning the transducer between measures. The bars represent the standard deviation of the repeated measurements. The average of the standard deviations is $\sigma_{M}=1.18$ ns. Figure 13b shows the CTOF on the *y*-axis, obtained by subtracting the no-load TOF from the measured TOF values; on the *x*-axis, there is the axial stress $\sigma =F_{L}/A$, where $F_{L}$ is the force measured by the reference load cell and *A* is the bolt cross-sectional area. Figure 13b also shows the theoretical behavior of CTOF vs. stress, obtained by inverting Equation (2), assuming the value U=3.0 for the ultrasonic constant [27] and E=200 GPa for Young’s modulus. Therefore, there is very good agreement between the theoretical behavior and the measured one. Figure 13c shows the measured change in the speed of sound due to the acousto-elastic effect, computed according to Equation (3):

(3) $$\Delta c_{M}=\frac{2(L_{C}+d)}{TOF_{L_{C}}+CTOF_{M}}-c_{0}$$

where $d=\frac{F_{L}L_{C}}{EA}$ is the bolt elongation computed from the load cell measure, assuming elastic deformation; $TOF_{L_{C}}=2L_{C}/c_{0}$ is the propagation time in the clamped section at no load; $CTOF_{M}$ is the measured CTOF, i.e., the same reported in Figure 13b. Figure 13c also shows the theoretical behavior of the change in the speed of sound vs. stress, computed as a function of stress σ according to Equation (4):

(4) $$\Delta c_{T}=\frac{2(L_{C}+d_{T})}{TOF_{L_{C}}+CTOF_{T}}-c_{0}$$

where $d_{T}=\sigma L_{C}/E$ is theoretical bolt elongation computed as a function of stress, assuming elastic deformation, and $CTOF_{T}=2d_{T}U/c_{0}$ is the theoretical CTOF computed as a function of stress. Please note that $CTOF_{T}$ is expressed as a function of constant $c_{0}$, while $c\left(\sigma \right)=c_{0}+\Delta c_{T}$ would be more correct instead; however, the use of $c_{0}$ streamlines Equation (4), introducing a negligible error since $c_{0}+\Delta c_{T}\approx c_{0}$.

## 4. Discussion

The research presented in this paper successfully demonstrated the feasibility of using a PMUT-based system for bolt preload monitoring. The system, which operates in pulse-echo mode, measures the Change in Time-of-Flight of acoustic waves to determine the preload of a bolt. Finite Element Method simulations were instrumental in optimizing the system design, considering factors such as transducer geometry, placement, and the presence of threads. The novel assembly process involving a polyurethane elastomer acoustic matching layer proved essential for effective dry-coupling of PMUTs to the bolt surface. Experimental validation of the system showed good signal-to-noise ratio and repeatability, with the experimental measurements aligning closely with theoretical predictions. In particular, to understand the capability of the system to measure bolt preload in different joint types and geometries, it is convenient to define a sensitivity figure by rearranging Equation (2):

(5) $$S=\frac{CTOF}{\sigma L_{c}}=\frac{2U}{cE}≃5.12\times 10^{-3}\frac{\left[\mathrm{ns}\right]}{\left[\mathrm{MPa}][\mathrm{mm}\right]}$$

where *S* represents how much CTOF is expected per unit axial stress and per unit clamped length. Considering a typical joint with long tens of millimeters and designed to work in the hundreds of MPa, the typical CTOF full scale is tens of nanoseconds. Since the experimentally measured CTOF standard deviation is $\sigma_{m}=1.18$ ns, which is much smaller than the CTOF full scale, we can state that the system is suitable for accurate measurements in most joints.

This work contributes to the field of structural health monitoring by introducing a low-cost, low-power, and highly accurate method with great potential for continuous bolt preload assessment. The PMUT-based system’s ability to be retrofitted onto existing joints with minimal effort further enhances its practicality and potential for widespread adoption. The research also advances the understanding of wave propagation in bolted joints, providing valuable insights that can inform future designs and improvements in ultrasonic testing technologies. By demonstrating the negligible impact of bolt threads on signal integrity and the benefits of using a soft solid mechanical coupling and acoustic matching layer, this study lays the groundwork for more robust and reliable monitoring systems. To better address the full development of a monitoring system, future work will include an investigation of aging effects during the whole lifetime of the bolt, data management, and investigation of the impact of temperature and its compensation.

## Data Availability

Dataset available on request from the authors.

## Abbreviations

The following abbreviations are used in this manuscript:

ADC	Analog-to-Digital Converter
ASIC	Application-Specific Integrated Circuit
AFE	Analog Front-End
CMOS	Complementary Metal-Oxide-Semiconductor
CTOF	Change in Time-of-Flight
FEM	Finite Element Method
MCU	Microcontroller Unit
MEMS	Micro-Electro-Mechanical Systems
NDT	Non-Destructive Testing
PMUT	Piezoelectric Micromachined Ultrasonic Transducer
RX	Receiver
SNR	Signal-to-Noise Ratio
TF	Transfer Function
TOF	Time of Flight
TX	Transmitter
UT	Ultrasonic Testing

## References

[1] Barrett R. (2005). Fastener Design Manual.

[2] Bickford J. (1995). An Introduction to the Design and Behavior of Bolted Joints.

[3] Oberg E., Jones F., Horton H., Ryffel H. (2004). Machinery’s Handbook.

[4] Lyndon B., Center J.S. (1998). Criteria for Preloaded Bolts.

[5] Fukuoka T., Takaki T. (1998). Mechanical behaviors of bolted joint during tightening using torque control. JSME Int. J. Ser. Solid Mech. Mater. Eng..

[6] Nikravesh S.M.Y., Goudarzi M. (2017). A review paper on looseness detection methods in bolted structures. Lat. Am. J. Solids Struct..

[7] Jhang K.Y., Quan H.H., Ha J., Kim N.Y. (2006). Estimation of clamping force in high-tension bolts through ultrasonic velocity measurement. Ultrasonics.

[8] Koshti A.M., Shull P.J. (2015). Ultrasonic measurement and monitoring of loads in bolts used in structural joints. Proceedings of the Structural Health Monitoring and Inspection of Advanced Materials, Aerospace, and Civil Infrastructure 2015.

[9] Liu Y., Liu E., Chen Y., Wang X., Sun C., Tan J. (2020). Measurement of fastening force using dry-coupled ultrasonic waves. Ultrasonics.

[10] Villa M., Ornaghi D., Boniolo I.E. (2021). A Sensorized Clamping Element. Patent Application.

[11] Tokbo Tokbo S.r.l. Website. https://tokbo.it.

[12] Jung J., Lee W., Kang W., Shin E., Ryu J., Choi H. (2017). Review of piezoelectric micromachined ultrasonic transducers and their applications. J. Micromech. Microeng..

[13] Przybyla R.J., Shelton S.E., Lee C., Eovino B.E., Chau Q., Kline M.H., Izyumin O.I., Horsley D.A. Mass Produced Micromachined Ultrasonic Time-Of-Flight Sensors Operating in Different Frequency Bands. Proceedings of the 2023 IEEE 36th International Conference on Micro Electro Mechanical Systems (MEMS).

[14] Gan T., Hutchins D., Billson D., Schindel D. (2001). The use of broadband acoustic transducers and pulse-compression techniques for air-coupled ultrasonic imaging. Ultrasonics.

[15] Savoia A.S., Matera R., Quaglia F., Ricci S. A feasibility study of a PMUT-based wearable sensor for the automatic monitoring of carotid artery parameters. Proceedings of the 2021 IEEE International Ultrasonics Symposium (IUS).

[16] Ji W., Liu L., Xing Z., Zhang D., Wang Y., Chen L., Chen Y., Sun X., Du Y. (2021). Total-Focus Ultrasonic Imaging of Defects in Solids Using a PZT Piezoelectric Micromachined Ultrasonic Transducer Array. IEEE Trans. Ultrason. Ferroelectr. Freq. Control.

[17] Abdalla O.M.O., Massimino G., Savoia A.S., Quaglia F., Corigliano A. (2022). Efficient Modeling and Simulation of PMUT Arrays in Various Ambients. Micromachines.

[18] Abdalla O.M.O., Massimino G., Quaglia F., Passoni M., Corigliano A. (2023). Pmuts arrays for structural health monitoring of bolted-joints. Micromachines.

[19] Savoia A.S., Giusti D., Prelini C., Chang P., Leotti A., Lee J., Koh Y., Ferrera M. Performance Analysis of Wideband PMUTs: A Comparative Study Between Sol-Gel PZT, PVD PZT, and 15% ScAlN-Based Arrays Through Experimental Evaluation. Proceedings of the 2023 IEEE International Ultrasonics Symposium (IUS).

[20] Picco A., Ferrarini P., Pedrini C., Cimmino A., Vinciguerra L., Vimercati M., Barulli A., Lazzari C.M., Vigna B., Ferrari P., Villa F.F., Lasalandra E., Zerbini S. (2022). Piezoelectric Materials for MEMS. Silicon Sensors and Actuators: The Feynman Roadmap.

[21] Savoia A.S., Giusti D., Prelini C., Saccher M., Rashidi A., Leotti A., Giagka V., Ferrera M. Evaluating the Influence of PMUT Mechanical Support Properties on Power Conversion Efficiency in Ultrasonically Powered Implants. Proceedings of the 2023 IEEE International Ultrasonics Symposium (IUS).

[22] COMSOL Introduction to the Elastic Waves, Time Explicit Interface. https://www.comsol.com/blogs/introduction-to-the-elastic-waves-time-explicit-interface.

[23] Amar A.B., Cao H., Kouki A.B. (2017). Modeling and process design optimization of a piezoelectric micromachined ultrasonic transducers (PMUT) using lumped elements parameters. Microsyst. Technol..

[24] Savoia A.S., Mazzanti A., Ottaviani S., Novaresi L., Malcovati P., Ghisu D.U., Bonizzoni E., Terenzi M., Quaglia F. A 4-channel Fully Integrated Ultrasound Imaging Front-End Transceiver for 1-D PMUT Arrays. Proceedings of the 2022 IEEE International Ultrasonics Symposium (IUS).

[25] Novaresi L., Malcovati P., Mazzanti A., Bonizzoni E., Terenzi M., Ottaviani S., Ghisu D.U., Quaglia F., Savoia A.S. A PMUT Transceiver Front-End with 100-V TX Driver and Low-Noise Voltage Amplifier in BCD-SOI Technology. Proceedings of the ESSCIRC 2022—IEEE 48th European Solid State Circuits Conference (ESSCIRC).

[26] Chen C., Pertijs M.A.P. (2021). Integrated Transceivers for Emerging Medical Ultrasound Imaging Devices: A Review. IEEE Open J. Solid-State Circuits Soc..

[27] Pärlstrand A. (2018). Ultrasonic measurement and analysis of screw elongation. MSc. Thesis.

[28] Oralkan O., Ergun A.S., Cheng C.H., Johnson J.A., Karaman M., Lee T.H., Khuri-Yakub B.T. (2003). Volumetric ultrasound imaging using 2-D CMUT arrays. IEEE Trans. Ultrason. Ferroelectr. Freq. Control.

[29] Oud T. (2017). Elastic Wave Simulation for Buffer Rod Tapering. Bachelor’s Thesis.

[30] Froeling H.A.J. (2017). Causes of Spurious Echoes by Ultrasonic Wave Simulation. BSc. Thesis.

[31] Ono Y., Jen C.K., Moisan J.F., Su C.Y. (2005). Aluminum buffer rods for ultrasonic monitoring at elevated temperatures. IEEE Trans. Ultrason. Ferroelectr. Freq. Control.

[32] Walton K., Skliar M. Echogenic Segmentation for Ultrasonic Measurements of Spatially Distributed Properties in Solids. Proceedings of the 2023 IEEE International Ultrasonics Symposium (IUS).

[33] Savioja L., Svensson U.P. (2015). Overview of geometrical room acoustic modeling techniques. J. Acoust. Soc. Am..

[34] Chen J., Fei C., Lin D., Gao P., Zhang J., Quan Y., Chen D., Li D., Yang Y. (2022). A review of ultrahigh frequency ultrasonic transducers. Front. Mater..

[35] Singh R., Singh R. (2020). 6—Ultrasonic testing. Applied Welding Engineering.

[36] Kim Y.H., Choi J.W. (2013). Radiation, Scattering, and Diffraction. Sound Visualization and Manipulation.

